# Transaneurysmal Occlusion of Complicated Common Femoral Artery Pseudoaneurysms Using the Angio-Seal Closure Device—A Promising Technique

**DOI:** 10.1007/s00270-022-03332-7

**Published:** 2022-12-16

**Authors:** Timo A. Auer, Uli Fehrenbach, Giovanni F. Torsello, Federico Collettini, Gero Wieners, Rolf W. Günther, Bernhard Gebauer

**Affiliations:** 1 Department of Radiology, Campus Virchow-Klinikum, Charité–Universitätsmedizin Berlin, Augustenburger Platz 1, 13353 Berlin, Germany; 2 Berlin Institute of Health (BIH), Anna-Louisa-Karsch-Straße 2, 10178 Berlin, Germany; 3 Institute for Diagnostic and Interventional Radiology, University Medical Center, Göttingen, Robert-Koch-Straße 40, 37075 Göttingen, Germany

**Keywords:** Pseudoaneurym, Angio-Seal, Common femoral artery, Closure device, TAVI

## Abstract

**Purpose:**

Pseudoaneurysm (PSA) developing after catheter examinations is one of the most frequent vascular complications and a nonsurgical technique with utmost low risk of complications is warranted. Our aim was to investigate the technical feasibility, success, and safety of transaneurysmal occlusion of complicated post-interventional common femoral artery (CFA) PSA using the Angio-Seal Closure Device (ASCD) and a technique that we describe as the transaneurysmal (TA) maneuver.

**Material and Methods:**

We used the *Angio-Seal* (*Terumo*, Tokyo, Japan) Closure System to manage complicated PSAs in patients who would otherwise have needed surgery after failure of all conservative therapies. The TA maneuver was performed in 14 consecutive patients from July 2021 to July 2022. After ultrasound-guided puncture of the PSA close to its neck, the CFA was entered radiographically with micro-guidewires, and the neck of the PSA was closed with the ASCD after changing the sheaths and wires. All patient had to wear a pressure dressing until the next day, when successful closure was verified by sonography.

**Results:**

All procedures were performed with technical success and without any complications. No patient had to undergo surgery. All sonographies on the next day confirmed complete absence of perfusion within the PSA and normal flow conditions of the CFA and vessels below.

**Conclusion:**

The TA maneuver a promising minimally invasive procedure for closing complicated PSA of the CFA after catheter examination.

## Introduction

Postcatheterization pseudoaneurysm (PSA) is one of the most common vascular complications of angiographic procedures and has been known for decades [1]. The incidence of PSAs of the common femoral artery (CFA) is low when a small-caliber device and an adequate technique are used. However, PSA after use of large-caliber devices remains a problem and may cause relevant morbidity [2].

Reported general incidences of PSAs, e.g., after cardiac interventions via the CFA, range from 0.2 to 8%, which may appear to be small, but considering the number of cardiac catheterizations performed worldwide, PSA is a relevant complication [3–7]. In the US, for example, an estimated 1 million cardiac catheter examinations are carried out annually; assuming an incidence of 1%, about 10,000 PSAs develop each year in the US alone.

Of course, not all of these PSAs persist. Some resolve spontaneously or after longer local compression. In addition, various other techniques are available including ultrasound- or stethoscope-guided compression, direct thrombin injection, embolization with coils or n-butyl cyanoacrylate, and stent graft placement [1, 6, 8–12]. To date, thrombin injection seems to be the nonsurgical technique of choice with different success but low complication rates reported [1, 6, 13, 14]. Nevertheless, a subset of patients still has to be treated surgically. As these patients are often multimorbid and on therapeutic anticoagulation the risk of perioperative complications is not to be underestimated. A nonsurgical option for conservative PSA treatment is highly desirable for these patients.

The transaneurysmal (TA) maneuver using the Angio-Seal Closure Device (ASCD—*Angio-Seal-System, Terumo, Tokyo, Japan*) is a young and promising technique to seal complicated postcatheterization CFA PSA. Nevertheless, apart from a few case reports, there is hardly any data published on this subject. The publication of a larger case series from a single institution may help this method to be spread and established [15, 16].

## Material and Methods

### Patients

The TA maneuver was performed in 14 consecutive patients from July 2021 to July 2022. In all patients, the TA maneuver was performed after failure of conservative or other minimally invasive measures such as prolonged pressure banding, improvement in the anticoagulation (if possible) or thrombin injection, thus sparing the patients an operation. As the technique was newly established, patient selection was individualized.

### Procedure

**Fig. 1:**
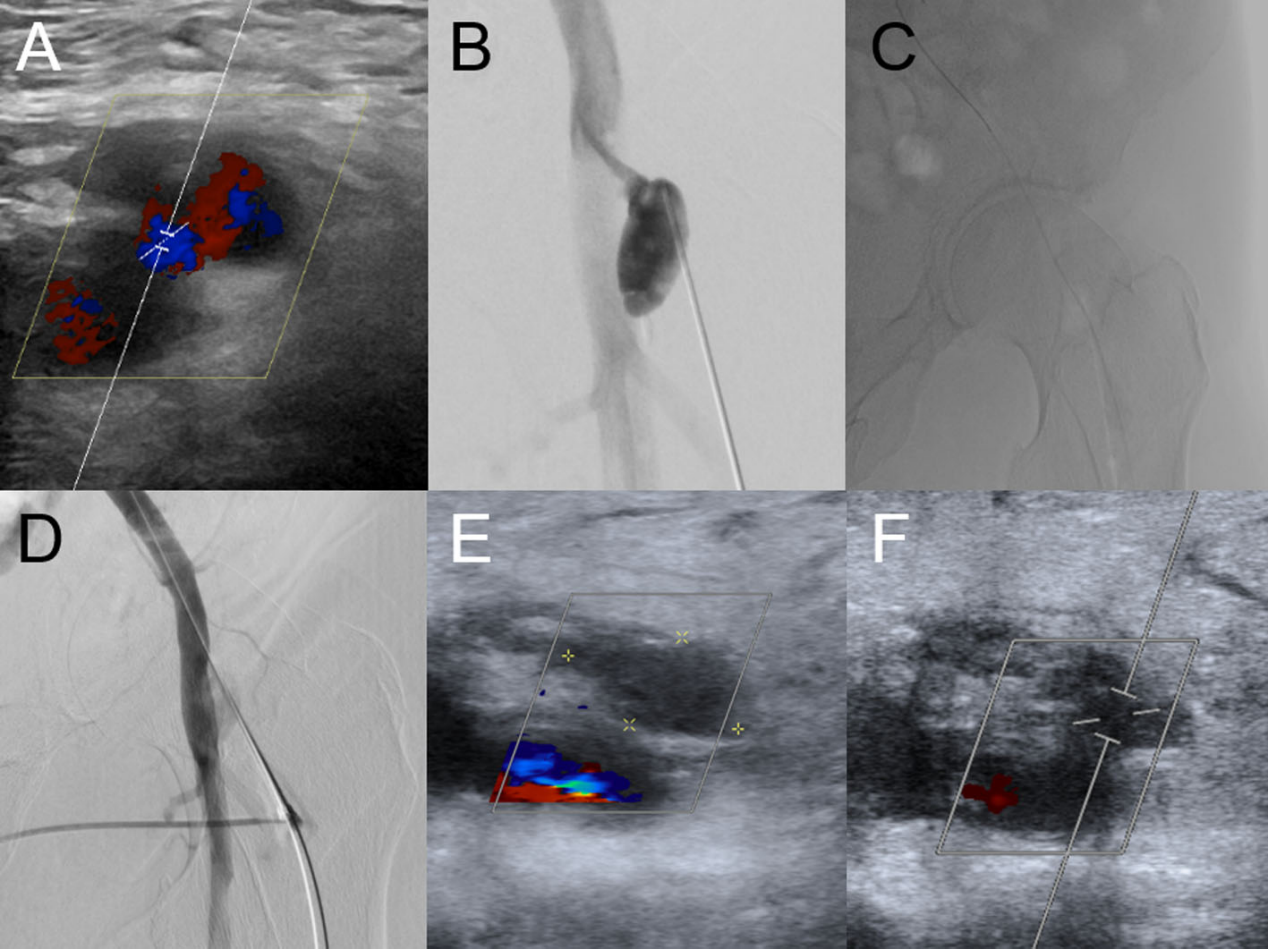
Well-perfused PSA of the CFA in a 91-year-old patient (Patient No. 3) after TAVI. **A** Sonography showing persistent perfusion after single thrombin injection. **B** Puncture of the PSA close to its neck and DSA via the connecting catheter to visualize the patient’s vascular anatomy. **C** Correct probing and positioning of the microwire in the pelvic axis. **D** Insertion of a 5 F sheath after changing to a 0035’ wire and DSA showing only residual perfusion of the PSA, confirming correct position. **E** and **F** Sonography on the next day shows nonperfused PSA the next day

**Fig. 2:**
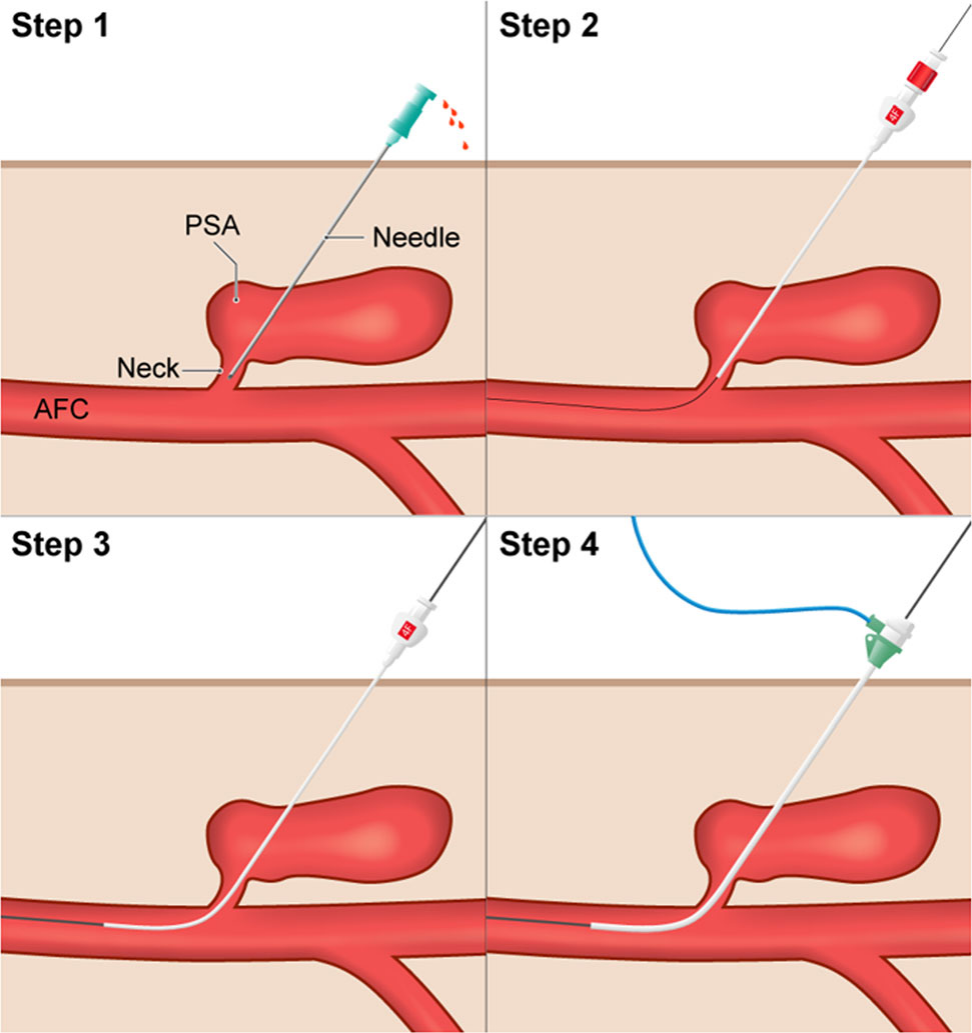
Shows our own schematic illustration of the procedure using MERIT Medical's Coaxial Mini Access Kit (MAK—Merit, South Jordan, USA) and a standard 5F sheath (in domo: TERUMO Radifocus® Introducer II (Terumo, Tokyo, Japan) Step 1* – Puncturing the PSA; Step 2* – Inserting the mini-sheath; Step 3* – Changing the wires; Step 4 – Inserting the sheath

**Fig. 3:**
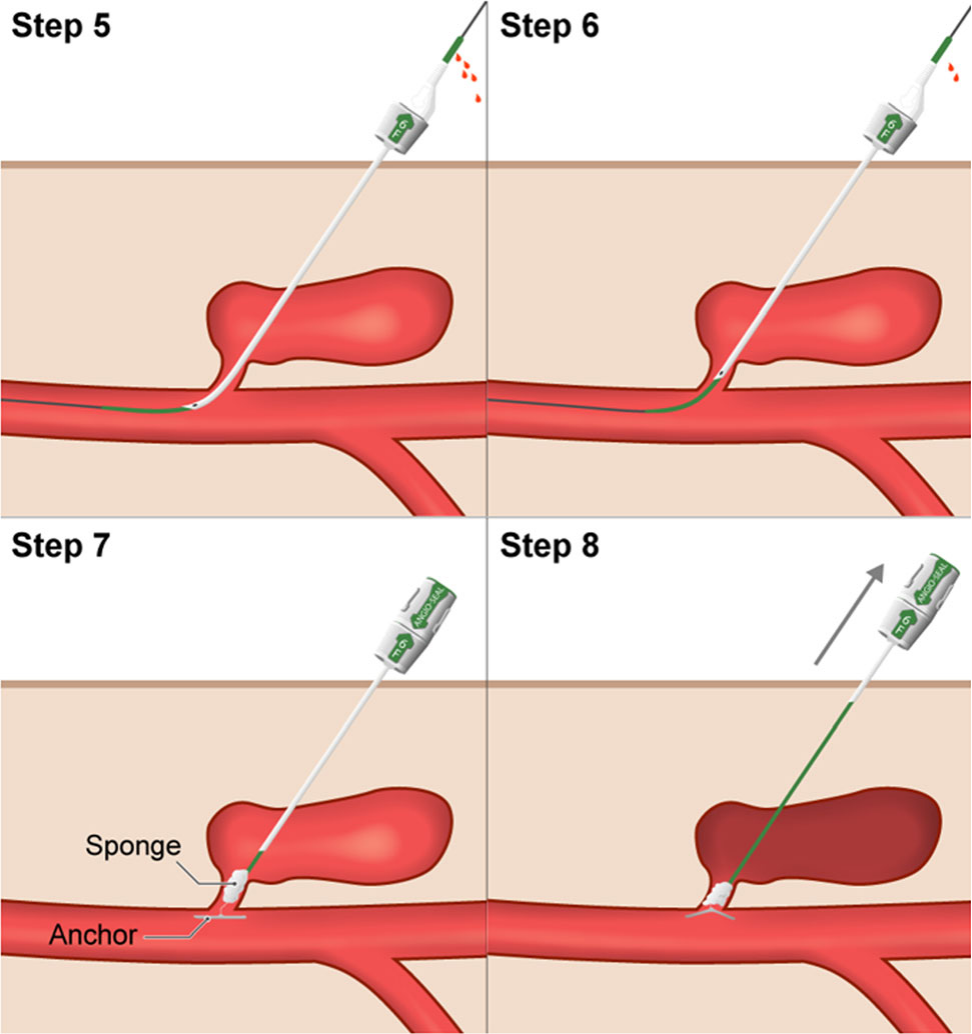
Also own schematic drawings: Step 5*—Locating the artery; Step 6* – Locating the PSA neck; Step 7*—Setting the anchor; Step 8* – Placing the sponge

After sterile draping and local anesthesia, the PSA is punctured directly as close as possible to its neck using a 21 G 7 cm needle and sonographic guidance using a linear transducer probe. (Figs. 1b, 2, Step 1– Puncturing the PSA). The 21 G needle is then connected to a short extension line and digital subtraction angiography (DSA) is performed to assess the needle location and its relationship to the neck and to choose an appropriate microwire (0.014″-0.018″) to probe it (Fig. 1b). Once the neck and CFA have been probed, the small 4F (1.3 mm) × 10 mm coaxial introducer sheath can be inserted (Fig. 2, Step 2–Inserting the mini-sheath). Once correct positioning has been proven by fluoroscopy with contrast medium administration, the dilator can be removed and the wires are switched from a micro- to a macrowire (Fig. 2, Step3– Changing the wires). We have come to prefer the 0.035″ J-wire (145 cm, 3 mm—Fixed Core Wire Guide; Safe -T-J; *COOK Medical*, Bloomingtion, USA), over which we then insert a standard 5 F sheath (*TERUMO Radifocus® Introducer II* (*Terumo*, Tokyo, Japan) (Fig. 2, Step 4–Inserting the sheath). With the sheath in place, DSA is performed to assess residual perfusion: If the sheath catheter seals the neck adequately, the chances of the maneuver being successful are extremely high. However, even in cases where the PSA is still perfused with the 5F sheath in place (Fig. 1d), our experience so far suggests that final closure can be accomplished with the TA maneuver. Although this step is useful to get a sense of the size of the neck is to discuss whether you can omit the step. The final elimination of the PSA is accomplished with the ASCD closure system. So far, we have used a 6F ASCD in all our patients. Closing the aneurysm with the ASCD is the crucial step and is different from closing a normal vessel. The aim is to place the anchor directly at the perforation site so that the collagen polymer is released into the PSA close to its neck. As for normal occlusion, the Angio-Seal locator and its lock system are first advanced into the vessel until blood comes out of the side hole at the proximal end of the locator (Fig. 3, Step 5-Locating the artery). Now, however, the system is not retracted into the subcutaneous fat, but into the PSA neck. The crucial question is to know when the locator tip has left the artery and is within the aneurysm neck and has not been withdrawn too far into the aneurysm sac. This is the case when the amount of blood coming out of the side hole is reduced from spurting to trickling (Fig. 3, Step 6–Locating the PSA neck). Once the stream of blood is reduced to trickling, the system can be advanced in toto by about 1–2 cm back into the artery. Now the locator and wire can be removed while the introducer is held in position at a 45° angle. Next, the Angio-Seal device can be inserted into the sheath to release the anchor (Fig. 3, Step 7–Setting the anchor). Ideally, the anchor then pulls into the neck. With the usual pull-back mechanism, only the collagen sponge can be pulled into the neck and the aneurysm (Fig. 3, Step 8–Placing the sponge).

### Evaluation

Data were collected from our clinical charts. Technical success was defined as complete absence of perfusion from the PSA and normal flow conditions in the CFA and vessels below in an ultrasound examination performed the next day.

## Results

**Fig. 4:**
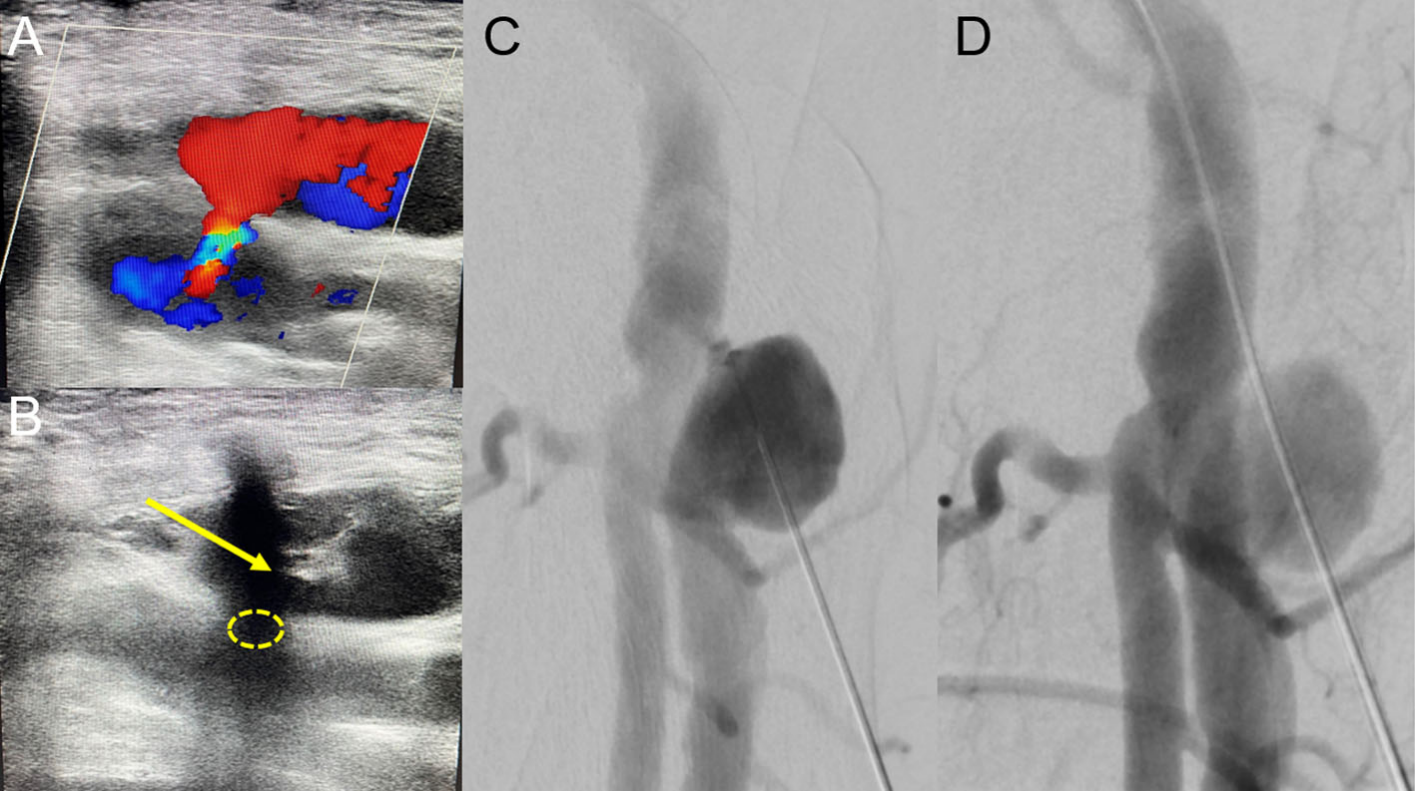
Huge PSA in a 94-year-old patient (Patient No. 2) after TAVI and a successfully performed TA maneuver on day 6 after the intervention. **A** Color-coded duplex sonography showing a huge PSA with a narrow neck and strong and turbulent perfusion. **B** Puncturing of the PSA with the needle tip (yellow arrow) close to the neck (dashed yellow circle). **C** Consistent with ultrasound (**B**), DSA confirms direct puncture of the PSA neck. **D** Despite successful and correct insertion of the 5F sheath through the PSA neck, there is persistent perfusion of the PSA. Nevertheless, sonography on the next day shows complete absence of perfusion from the PSA following the TA maneuver

**Fig. 5:**
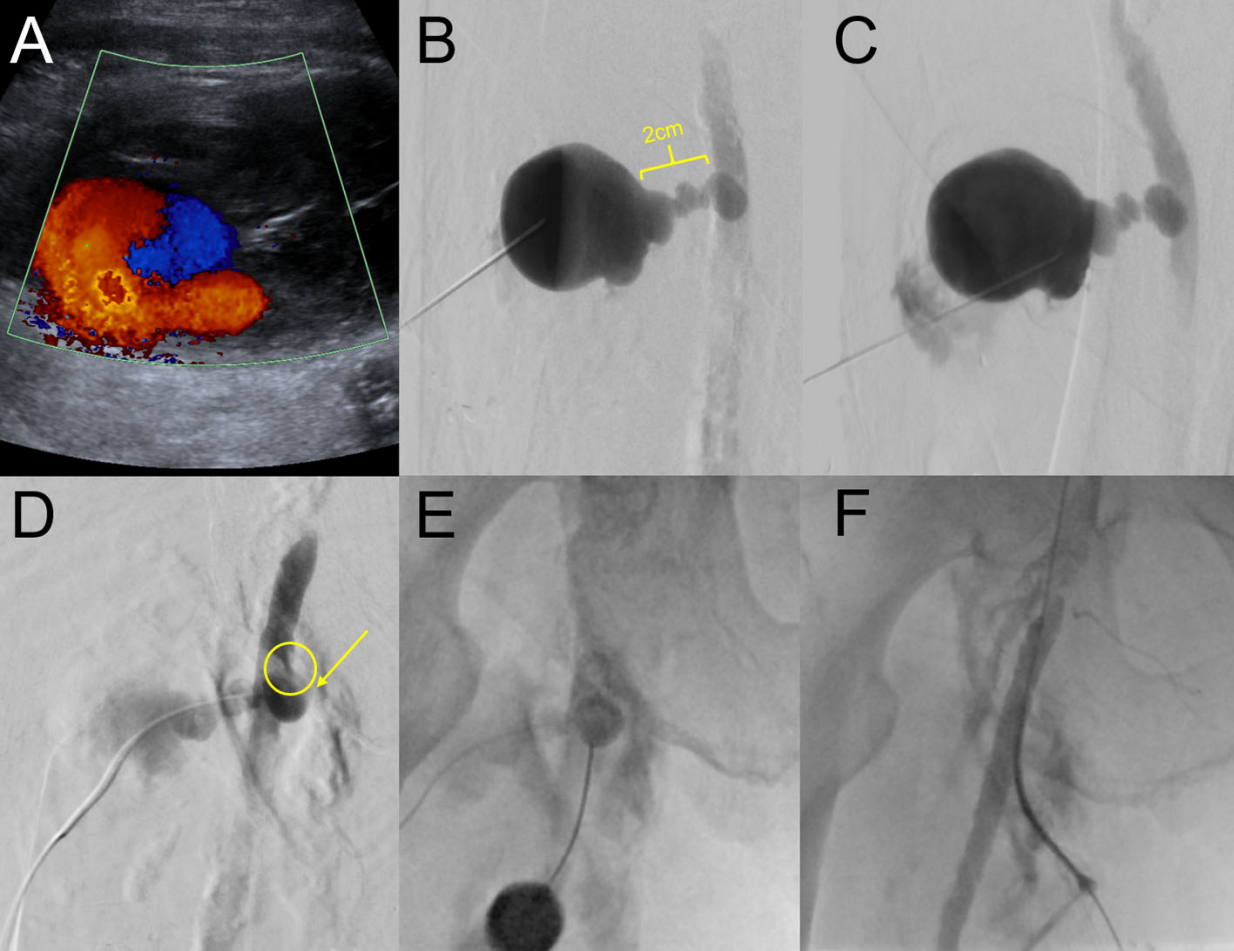
Gigantic PSA in a 75-year-old patient (Patient No. 4) after TAVI, which was successfully closed by the TA maneuver 6 days after the intervention. **A** Sonography of the gigantic PSA in the right groin. **B** Initial puncture far away from the PSA neck without any chances to probe the neck via a microwire. **C** Second puncture closer to the multilobulated neck but still without any realistic chances to reach the neck and the pelvic axis. **D** Nevertheless, via the second puncture, it was possible to position a MAK mini-sheath close to the neck. **E** Parallel fluoroscopic puncture and visualization of the PSA via the mini-sheath while rotating the tube made it possible to directly puncture into the neck. **F** Next, a 5F sheath was inserted successfully, and the TA maneuver could be performed. Although the PSA was gigantic in size, its small and lobulated neck promised good chances of success. Successful closure was confirmed by complete absence of perfusion from the PSA in sonography the day after

Technical success was 100% and no complications occurred. No patients had to undergo surgery. All sonographies on the next day showed a completely nonperfused PSA and normal flow in the CFA and vessels below, and the patients could be discharged from hospital. As far as we can tell from our database, none of the patients experienced associated complications in the groin during the procedure summarizes patient characteristics and treatment-related data for all patients (Table 1). Figures 4 and 5 illustrate further interesting patient examples (Figs. 4 and 5).

## Discussion

The transaneurysmal approach using the ASCD is a young and promising technique to seal complicated postcatheterization CFA PSA and may spare surgery, thus morbidity, and potentially saving costs. Over the last few years, other authors have already reported similar attempts using the ASCD [15, 16].

First proposed in 1986 by *Cope* and *Zeit,* thrombin injection has become the preferred nonsurgical technique for treating PSA [1, 13]. While published success rates differ, thrombin injection seems to be superior to ultrasound-guided compression and other techniques and has a low complication rate of 0–4% [6, 14]. Nevertheless, a subset of patients still has to be treated surgically. In our opinion, the TA maneuver can be performed in both in large PSA where thrombin injection may be unsuccessful and as a follow-up intervention after thrombin injection has failed.

Although technical success was 100% and no complications occurred in our patient population the technique is prone to the same complications reported in the literature for use of the ASCD. In a meta-analysis *Nikolsky *et al*.* reported of ASCD related complication rates of up to 2.5% (17). Overall, published data suggest that complications tend to occur in patients with calcified arteries. These experiences may help identify patients with post-interventional PSA who should not be treated with the TA maneuver in the first place.

Of course, the case series presented here has some limitations and we would like to highlight two of them. One is the small number of cases and the other is the incongruent patient selection, which is due to the fact that we are reporting our first experience with this technique. In conclusion is the TA maneuver a promising procedure for closing complicated PSA with a low probability of complications when performed thoroughly.

**Table 1:**
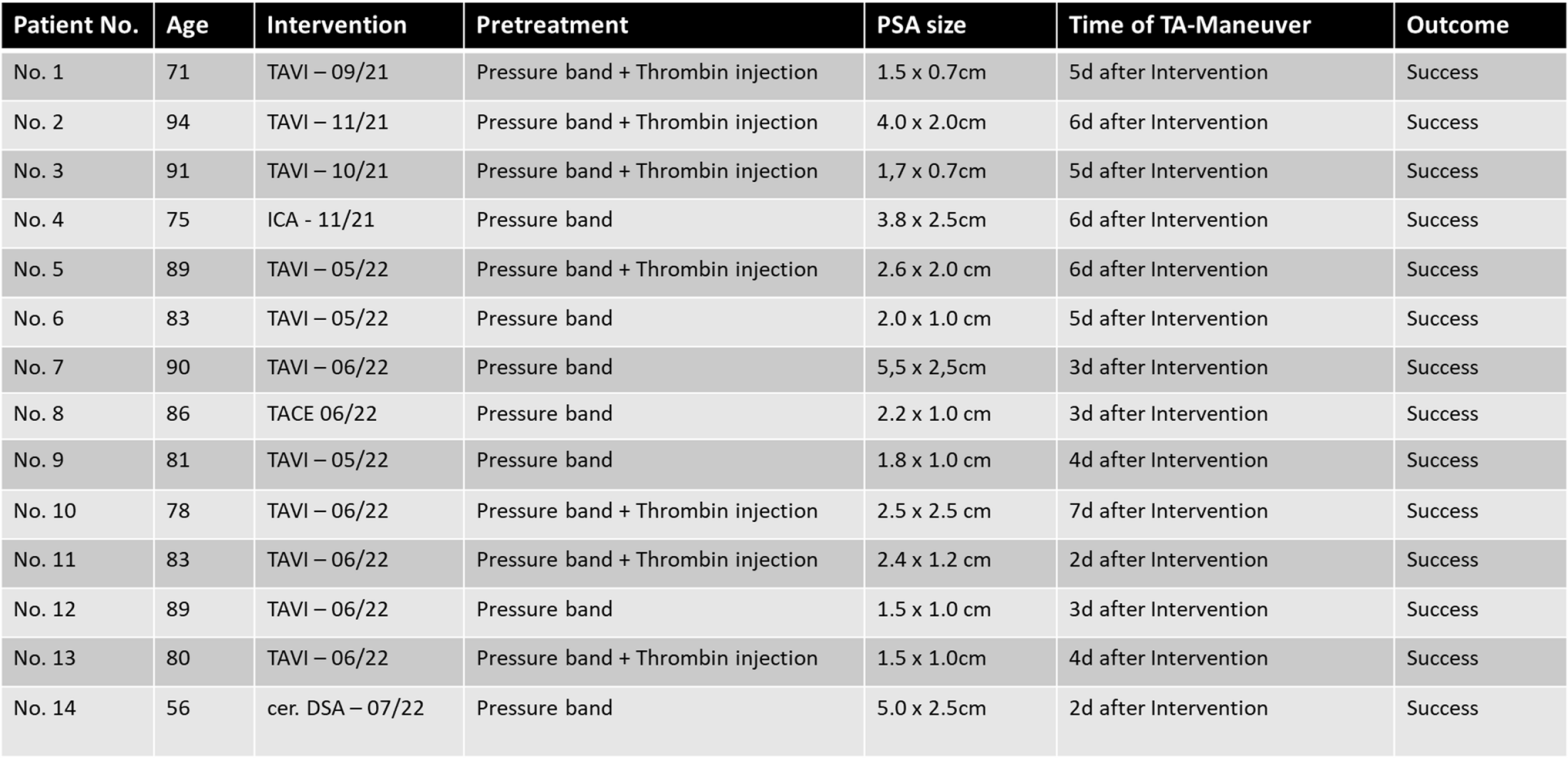
Patient characteristics
